# Improving data quality for three-dimensional electron diffraction by a post-column energy filter and a new crystal tracking method

**DOI:** 10.1107/S1600576722009633

**Published:** 2022-11-29

**Authors:** Taimin Yang, Hongyi Xu, Xiaodong Zou

**Affiliations:** a Department of Materials and Environmental Chemistry (MMK), Stockholm University, Svante Arrhenius väg 16 C, Stockholm, SE-10691, Sweden; Ecole National Supérieure des Mines, Saint-Etienne, France

**Keywords:** post-column energy filters, 3D electron diffraction, energy-filtered 3D ED, microcrystal electron diffraction, energy-filtered MicroED, HAADF, crystal tracking, structure determination

## Abstract

Three-dimensional electron diffraction (3D ED) has become an effective technique to determine the structures of submicrometre- (nanometre-)sized crystals. In this work, energy-filtered 3D ED was implemented using a post-column energy filter in both STEM mode and TEM mode [(S)TEM denoting (scanning) transmission electron microscope]. The setups for performing energy-filtered 3D ED on a Gatan imaging filter are described. The technique and protocol improve the accessibility of energy-filtered 3D ED post-column energy filters, which are available in many TEM laboratories. In addition, a crystal tracking method in STEM mode using high-angle annular dark-field imaging is proposed. This method enables the user to monitor the crystal position while collecting 3D ED data at the same time, allowing a larger tilt range without foregoing any diffraction frames or imposing extra electron dose. In order to compare the differences between energy-filtered and unfiltered 3D ED data sets, three well known crystallized inorganic samples have been studied in detail. For these samples, the final *R*_1_ values improved by 10–30% for the energy-filtered data sets compared with the unfiltered data sets, and the structures became more chemically reasonable. Possible reasons for improvement are also discussed.

## Abstract 2

Zero-loss energy-filtered 3D electron diffraction experiments are performed using a post-column energy filter, and both data quality and structures are improved after energy filtration. A novel crystal tracking method is developed, based on a live high-angle annular dark-field image stream. This method can track crystals to achieve high-completeness data sets without adding any additional dose or removing any frames.

## 1. Introduction

Over the past three decades, 3D electron diffraction (3D ED) has been developed into a reliable technique for structure determination, which is complementary to single-crystal X-ray diffraction (XRD), powder XRD and cryoEM single-particle analysis. The development of 3D ED as a method for structure determination is pioneered by electron diffraction tomography (EDT) (Kolb *et al.*, 2007 ▸) and rotation electron diffraction (Wan *et al.*, 2013 ▸), which utilize stepwise rotation around a single axis. More recently, new protocols such as continuous rotation microcrystal electron diffraction (Nan­nenga *et al.*, 2014 ▸; Rodriguez *et al.*, 2015 ▸; Jones *et al.*, 2018 ▸; Lanza *et al.*, 2019 ▸), fast-EDT (Gemmi *et al.*, 2015 ▸; Plana-Ruiz *et al.*, 2020 ▸) and continuous rotation electron diffraction (Cichocka *et al.*, 2018 ▸) have been developed for determining crystal structures of beam-sensitive materials. These are based on continuously rotating the crystal at a constant speed while collecting ED patterns at the same time. These setups have been widely applied in the structural determination of zeolites (Gemmi *et al.*, 2015 ▸; Seo *et al.*, 2018 ▸; Ge *et al.*, 2022 ▸), metal–organic frameworks (Wang *et al.*, 2018 ▸; Ge *et al.*, 2021 ▸), small organic molecules (Gruene *et al.*, 2018 ▸; Yang *et al.*, 2021 ▸) and proteins (Nederlof *et al.*, 2013 ▸; Clabbers *et al.*, 2017 ▸, 2021 ▸; Xu *et al.*, 2018 ▸).

One of the issues for ED is inelastic scattering, which increases the background in diffraction patterns. The inelastically scattered electrons can be removed by an energy filter. Gemmi & Oleynikov (2013 ▸) reported energy-filtered EDT using an in-column energy filter, and they found that the structure determined from energy-filtered data sets was closer to the results of X-ray refinement (Gemmi & Oleynikov, 2013 ▸). Yonekura and coworkers also utilized an in-column filter and carried out a systematic investigation of charges in protein crystals (Yonekura *et al.*, 2015 ▸, 2018 ▸, 2019 ▸; Yonekura & Maki-Yonekura, 2016 ▸; Maki-Yonekura *et al.*, 2021 ▸). However, transmission electron microscopes (TEMs) are not commonly equipped with an in-column energy filter. The low availability of in-column filters limits the application of energy-filtered 3D ED. In comparison, post-column energy filters, such as the Gatan imaging filter (GIF) system, are more commonly installed on modern TEMs. Until now, no energy-filtered 3D ED experiments have been performed using a post-column energy filter, and no protocol has been established for these experiments. Besides, most of the samples studied in previous energy-filtered 3D ED experiments were protein crystals, which have strong inelastically scattered background and little dynamical scattering.

In addition, the crystal will drift as the stage continuously rotates during data collection, even if the mechanical eucentric height has been carefully adjusted. The crystal can even move out of the beam or the selective area aperture during data collection. Therefore, tracking is necessary to obtain 3D ED data over a large tilt range. Several methods have been introduced to solve this problem. Images of the crystal can be formed at a predefined interval during 3D ED data collection by defocusing the intermediate lens (Cichocka *et al.*, 2018 ▸; Yang *et al.*, 2022 ▸). This method can track the position of the crystal over a large tilt range at the cost of 5–10% of the ED frames, depending on the predefined interval. It is also possible to record a movie of the crystal in image mode to monitor its movement during rotation before collecting 3D ED data sets (Gemmi *et al.*, 2015 ▸; Plana-Ruiz *et al.*, 2020 ▸; Ruiz, 2021 ▸). The crystal can be tracked during data collection at the cost of excessive electron dose (the pre-recorded movie). Neither method is ideal.

Here, we implemented energy-filtered 3D ED in both scanning TEM (STEM) mode and TEM mode using a GIF, which is one of the most popular post-column energy filters. We showed that post-column energy filters can achieve similar performance to their in-column counterparts. Nowadays, many microscopes are equipped with a GIF, making the method developed in this work widely accessible to researchers. The main advantage of this method is the removal of inelastically scattered electrons, which might remove a portion of dynamically scattered electrons at the same time (Yonekura *et al.*, 2015 ▸). We performed energy-filtered 3D ED on inorganic crystals, in which fewer electrons were inelastically scattered and more electrons were dynamically scattered compared with protein samples. In addition, to improve data quality, we developed a tracking method based on monitoring the live HAADF image stream to avoid losing diffraction frames for imaging or applying additional dose on the crystal for imaging before 3D ED data collection. This method reduces the overall electron dose and provides the possibility to correct the crystal position during stage rotation and data collection. It can keep the target crystal or an area of interest in the beam during continuous rotation. The overall tilt range for a single data set can easily reach the maximum tilt range of the microscope (in our case ∼150°).

## 2. Experimental

### 2.1. Samples

Data sets from NaCl (Sigma–Aldrich), NH_4_H_2_PO_4_ (Sigma–Aldrich) and finned zeolite ZSM-5 crystals (Dai *et al.*, 2020 ▸) were collected. The samples were crushed in a mortar and then dispersed in ethanol. After ultrasonication for 5 min, a drop of suspension was delivered onto a lacey-carbon copper grid. For the ZSM-5 sample, filtered and unfiltered 3D ED data sets were collected from the same crystal for direct comparison. In order to control the potential influence of electron dose, the collection sequence of filtered and unfiltered data sets was changed for different crystals. Furthermore, we collected energy-filtered data sets with different slit sizes (100, 50, 25, 10 and 5 eV) from the same ZSM-5 crystal.

### 2.2. Data collection

All the data sets were collected on a Themis-Z double-aberration-corrected microscope. The microscope is equipped with a monochromator, used for reducing the energy spread of the electron beam. However, in our experiment, the monochromator was used for adjusting the electron dose. Thus, it is not an essential device for the protocol. A Fischione model 2020 tomography holder was used. ED patterns were collected on an UltraScan1000FX CCD camera installed behind a GIF Quantum 965 ER. The width of the energy slit was adjusted to 10 eV and zero-loss peak (ZLP) was selected in all experiments. Before each TEM session, the GIF needs to be aligned by the automatic alignment function in *Digital Micrograph* (*DM*, Gatan, Pleasanton, California, USA). After automatic alignment, the position of the slit was checked manually. Once the alignment of the GIF is completed, the current of all lenses in the projection system should not be changed. For example, if the camera length is changed, the position of the energy slit needs to be checked again. In fact, the position of the energy slit should be checked occasionally to avoid slit position shift during the data collection session.

#### 2.2.1. Data collection in STEM mode with crystal tracking

**Figure 1:**
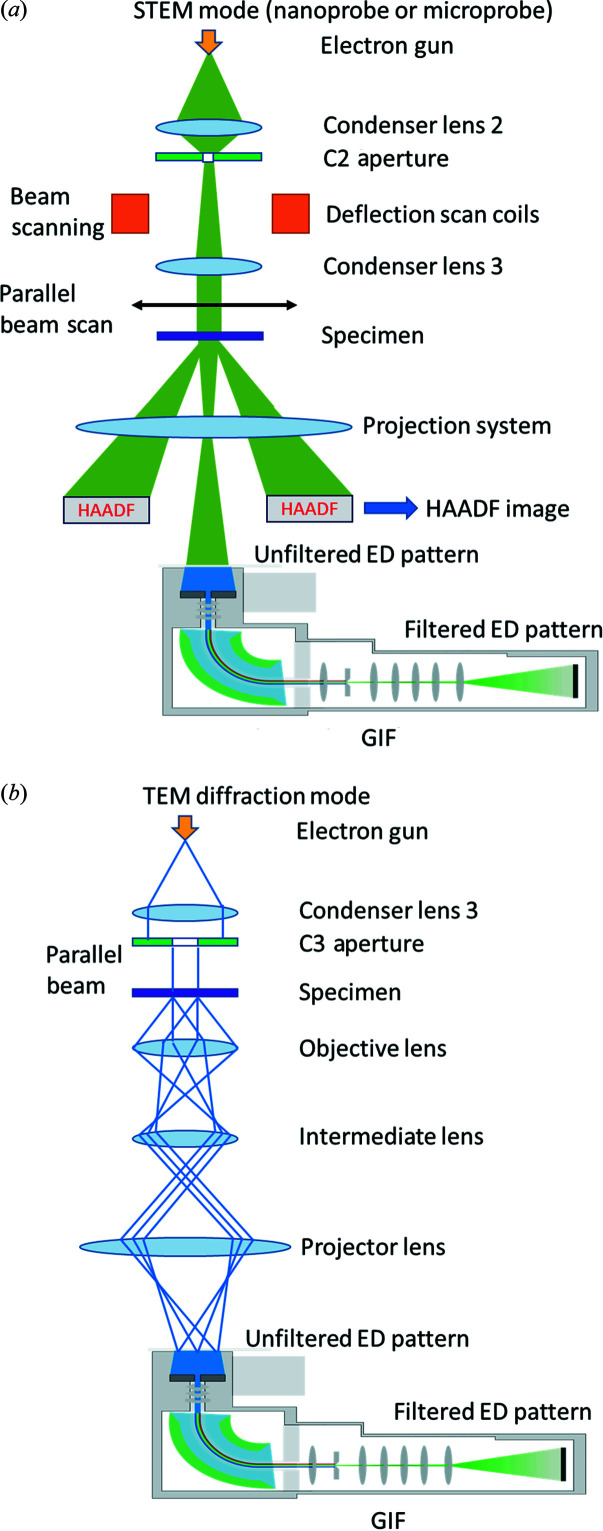
Schematics of energy-filtered 3D ED in (*a*) STEM mode (non-ray diagram) and (*b*) TEM mode (ray diagram). In STEM mode, crystal tracking using a STEM-HAADF image stream can be activated.

**Figure 2:**
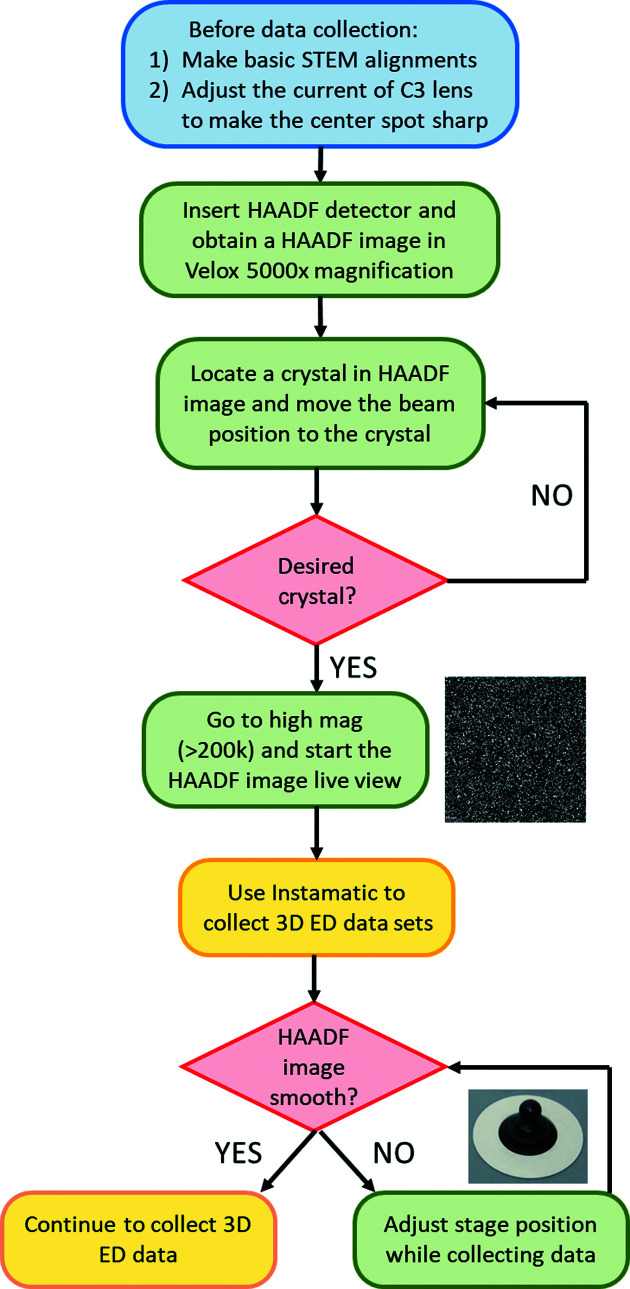
Workflow for STEM-HAADF crystal tracking while collecting 3D ED data sets using *Instamatic*. The blue, green and red boxes show steps that require human intervention. The yellow boxes show steps handled by the software. The STEM image at low magnification shows the positions where the user might find a desired crystal. The HAADF STEM image at high magnification monitors the position of the crystal when collecting 3D ED data sets.

A schematic of energy-filtered 3D ED in STEM mode is shown in Fig. 1 ▸(*a*). In order to track the crystal during continuous-rotation data collection, scanning transmission electron microscopy high-angle annular dark-field (STEM-HAADF) imaging is used. In this protocol, the microscope is operated in STEM mode and the beam is scanned over an area smaller than the crystal size in order to obtain a live HAADF image stream. The beam scan and HAADF image collection are controlled and visualized using the *Velox* software from Thermo Fisher Scientific. The electron beam is set to the nano-beam STEM mode. The size of the C2 aperture was 50 µm, which is the smallest C2 aperture available in our microscope. In the nano-probe STEM mode, the minimum convergence angle is 5 mrad. Quasi-parallel beam and sharp spots can be obtained by simply adjusting the defocus using the intensity knob, which changes the current of the C3 lens. If the C3 lens is not available, the currents of both the C1 and the C2 lenses need to be adjusted by free lens control in order to obtain a quasi-parallel beam (Plana-Ruiz *et al.*, 2020 ▸; Ruiz, 2021 ▸). The diameter of the parallel beam was around 240 nm. If the beam were kept stationary during stage rotation, the tilt range would be limited because the crystal can easily move out of the beam, especially at high tilt angles. Therefore, we scanned the beam during stage rotation. Due to the beam scan, the diffraction pattern will have small shifts caused by slight beam tilt if the scan area is large, which blurs the reflections. By keeping the scanning area relatively small (<200 nm), the movement of the diffraction spots can be minimized and the reflections will remain sharp. A schematic is presented in Fig. S1 of the supporting information to show the typical size of the beam and the size of the scanning area used in this work. Meanwhile, a HAADF detector was used to collect electrons scattered to high angles and form an image to track the position of the crystal. The scanning of the electron beam allows the formation of the HAADF image, while 3D ED data are collected over the entire illuminated area. The detailed workflow for STEM-HAADF crystal tracking is illustrated in Fig. 2 ▸. After the alignment of the microscope, a HAADF image was collected at low magnification (*e.g.* 5000×) with a parallel beam 240 nm in size for identifying the position of crystals, as shown in Fig. S2(*b*). Then the target crystal was moved to the centre and a HAADF image stream at high magnification (*e.g.* 910 000×) was displayed. At the same time, 3D ED data collection was started and the crystal began to rotate. During the rotation, if the target crystal starts to move out of the beam, one edge of the blurry HAADF image will change in contrast (*i.e.* become darker), as shown in Fig. S3(*a*). To position the crystal back in the beam, we need to move the crystal towards the dark area using the joystick, as shown in Fig. S3(*b*). Meanwhile, the remaining electrons not contributing to the STEM-HAADF imaging will go through the GIF and form energy-filtered diffraction patterns on the camera. As the crystal is continuously rotated, energy-filtered 3D ED data were collected with live crystal tracking by HAADF imaging.

The 3D ED data collection was performed using the data acquisition software *Instamatic* (Smeets *et al.*, 2017 ▸). The rotation speed of the goniometer was kept at 0.3° s^−1^ for NaCl and NH_4_H_2_PO_4_. For ZSM-5, the rotation speed was 0.6° s^−1^ to reduce the total electron dose. The exposure time was 1 s per frame for all data sets. The dose rate of the incident beam for all experiments was adjusted to 0.1 e Å^−2^ s^−1^. Movie S1 of the supporting information is provided to show the detailed data collection process.

### 2.3. Data processing and structure determination

After data collection, the data sets were processed using *XDS* for spot finding, unit-cell determination, indexing, space-group assignment, data integration, scaling, refinement and generating *SHELX* *hkl* files (Kabsch, 2010 ▸). Data statistics indicators provided in the output CORRECT.Lp file were used for data quality comparisons. Next, *SHELXT* (Sheldrick, 2015*b* ▸) was used for structure solution. Structure refinement and visualization of the structure models were performed using *SHELXL* (Sheldrick, 2015*a* ▸) and *ShelXle* (Hübschle *et al.*, 2011 ▸).

## 3. Results and discussion

### 3.1. Energy-filtered 3D ED in STEM mode

We first performed 3D ED experiments in STEM mode with a parallel beam (240 nm in diameter). With a nanometre-sized probe, we can keep the illuminated area as small as possible and avoid damaging nearby crystals. This setup can significantly increase the dose efficiency compared with the selected area ED (SAED) setup, which typically uses an electron beam of around 2–4 µm size and an SAED aperture of around 600–1000 nm diameter. By collecting energy-filtered 3D ED in STEM mode, there is no need to switch back and forth between imaging mode and diffraction mode, which is another advantage over operating in TEM mode. Because of inherent hysteresis of the projection lenses, the position of the ZLP will drift away from the original position when switching between STEM mode and imaging mode. As a result, the GIF alignment and position of the energy slit need to be adjusted. Therefore, most of the experiments reported in this work were performed in STEM mode.

#### 3.1.1. NaCl

Because of the simple structure and exceptional crystallinity of NaCl, it was chosen as a test sample to explore the experimental and refinement parameters and to show the improvement in data quality after energy filtration. Table S1 shows the *XDS* data-processing results. Benefiting from the STEM-HAADF tracking technique, the tilt range for all data sets can reach above 130°. Two data sets reached 150°, which is the maximum tilt range for the stage of the microscope. The tilt ranges for some data sets were smaller because of blockage by neighbouring crystals or the copper grid bars. The data were processed in space group *P*1 to ensure that the positions of the predicted spots match with the observed reflections. Improvement in the final *R*_1_ values was observed using energy-filtered 3D ED data, from 13.9 to 8.4% on average, as shown in Table S1.

Notably, during the refinement of the NaCl structure, we introduced the keyword ‘EXTI’ in the *SHELX* input file and found it had a huge impact on the final *R*_1_ values for all eight data sets. In the structure of NaCl, the positions of the Na and Cl atoms do not change during refinement. Only the atomic displacement parameters (ADPs) and scale factors are refined. Without the EXTI keyword, the final *R*_1_ value was around 20–35%, as shown in Table S1 of the supporting information. After using EXTI to weight the reflections, the final *R*_1_ value decreased sharply to around 9%. EXTI also provided noticeable improvement in the ADPs of the NaCl atoms, as shown in Fig. S4. Without the EXTI keyword, the ADP values of refinement structures were negative, while the ADP values became positive with EXTI.

#### 3.1.2. NH_4_H_2_PO_4_

**Figure 3:**
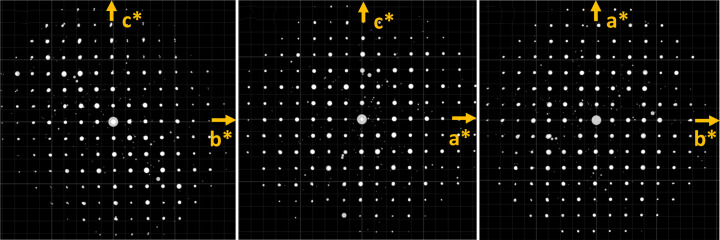
Typical 3D reciprocal lattices of NH_4_H_2_PO_4_ collected on the GIF reconstructed and visualized using the *REDp* software.

**Figure 4:**
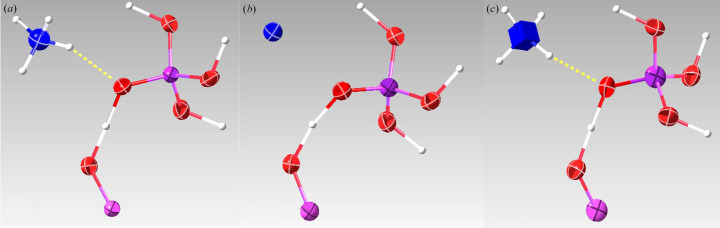
(*a*) Typical NH_4_H_2_PO_4_ crystal structure representation from a filtered data set. All hydrogen atoms were found and all the ADPs are reasonable after anisotropic refinement. (*b*), (*c*) Two typical NH_4_H_2_PO_4_ crystal structures obtained from unfiltered data sets. The refinement was unable to locate all the hydrogen atoms around the nitro­gen atom or to obtain reasonable displacement parameters for the nitro­gen atom. The dotted lines represent the hydrogen bond between the hydrogen atom and the oxygen atom. White – hydrogen; blue – nitro­gen; purple – phosphate; red – oxygen.

Next, we collected six NH_4_H_2_PO_4_ data sets (three unfiltered and three energy-filtered). Similar improvements in data quality and structure refinement statistics have been observed. For these crystals, the tilt range was around 140° and the final *R*_1_ value decreased from 12.4% (1.5%) to 10.2% (1.3%) on average when refined against energy-filtered 3D ED data with the EXTI keyword, as shown in Table 1 ▸. In addition, we compared refinements without EXTI. The final *R*_1_ value increased significantly compared with that using the EXTI keyword. However, the final *R*_1_ value still decreased from 22.9 to 20.2% on average after energy filtration. The results showed that energy filtration will reduce the final *R*_1_ value regardless of whether the EXTI keyword was applied. In all data sets, distortion of the 3D reciprocal lattice was visualized via the *REDp* software (Wan *et al.*, 2013 ▸). As shown in Fig. 3 ▸, the angles between the axes deviated from 90° (in the case of a tetragonal crystal system). The deviation was likely caused by the projection lenses or GIF lenses. The most common type of distortion in a TEM is elliptical distortion, which can be corrected by applying an affine transformation (Ångström *et al.*, 2018 ▸). Unfortunately, this type of distortion did not appear to be the dominant one, because when we corrected for the elliptical distortion, the unit-cell parameters did not improve. This indicates other types of distortion play an important role here. We also compared the structures refined against all six data sets. For energy-filtered data sets, all hydrogen atoms in the structures could be located and the bond angles between hydrogen atoms and their adjacent atoms were reasonable. After anisotropic refinement, all ADPs were positive and the shapes of the ellipsoids were chemically sensible, as shown in Fig. 4 ▸(*a*). As a proof of structural improvement, we show the structures refined with anisotropic ADPs against unfiltered data sets in Figs. 4 ▸(*b*) and 4(*c*) for comparison. In the refinement results for unfiltered data sets we were unable to find the hydrogen atoms around the nitro­gen atom or obtain reasonable displacement parameters for the nitro­gen atom.

#### 3.1.3. Evaluation of the stability of the new tracking procedure

We used NH_4_H_2_PO_4_ data sets as examples to evaluate the stability of the HAADF-STEM crystal tracking protocol. The normalized scaling factors for each ED frame (SCALE in file INIT.Lp) can determine the variation of incident beam flux and diffraction volume during data collection. When the crystal moves out of the beam scanning area, the corresponding diffracted intensities will be lower and a higher scale factor needs to be applied to that frame. Fig. S5 shows the scale factor data sets collected with the tracking method for three NH_4_H_2_PO_4_ crystals. The plot of the scale factor revealed a very smooth and slow variation profile over the whole tilt range, indicating that the diffraction volume of the crystal was relatively stable and the crystal stayed within the scanning area during tilting.

#### 3.1.4. ZSM-5

**Figure 5:**
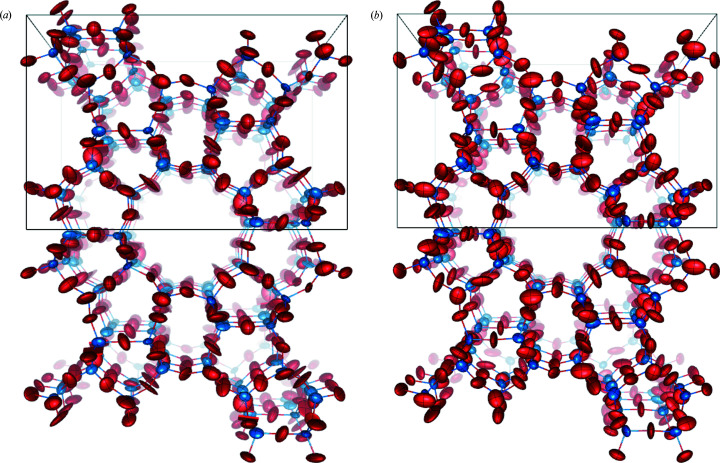
Refined structures of ZSM-5 crystal 1 from (*a*) unfiltered and (*b*) filtered data sets, showing ADPs for the Si and O atoms at the 60% probability level along the *b* axis. Red – oxygen atoms; blue – silicon atoms. The structure from the unfiltered data set contained many unreasonable ADPs; some became negative while the ADPs of the structure from the filtered data set were reasonable and closer to isotropic.

Compared with porous material, NaCl and NH_4_H_2_PO_4_ crystals have relatively simple structures and are robust against electron beam damage. Therefore, we used finned ZSM-5 as an example to show the improvements of data quality and structure determination in complex and beam-sensitive materials. Movie S2 was recorded to provide a visual comparison between filtered and unfiltered data sets. A comparison between the structures of finned ZSM-5 refined against filtered and unfiltered 3D ED data is shown in Fig. 5 ▸. We collected filtered and unfiltered data sets from the same crystal. In order to eliminate the effect of beam damage, for crystal 1, we first collected unfiltered data and then filtered data. For crystal 2, we reversed the data collection sequence and collected energy-filtered data first. The structure refined against unfiltered 3D ED data [Fig. 5 ▸(*a*)] contained negative ADPs (shown as thin rectangles) and some ADPs became very thin ellipsoids. The oscillation direction of some ellipsoids was along the bond, which is chemically unreasonable. In contrast, the structure refined against filtered data sets was improved significantly. The ADPs were very reasonable and no negative ADPs were observed, as shown in Fig. 5 ▸(*b*). The data statistics and refinement results were summarized in Table 2 ▸. The *I*/σ and CC_1/2_ values were improved in the energy-filtered data, and the final *R*_1_ value also decreased from 0.264 to 0.243 for crystal 1, and from 0.233 to 0.197 for crystal 2. Without the EXTI keyword, the final *R*_1_ value for crystal 1 still dropped from 0.300 to 0.275 after energy filtering was applied and crystal 2 showed a similar trend. In addition, the average deviation in atomic positions compared with the X-ray reference structure is shown in Table S2. The averaged deviations of both the Si and the O atoms were reduced by around 0.01 Å using the energy-filtered data. We also compared the bond lengths in filtered and unfiltered structures with the reference structure, and found that the unfiltered structure has some exceptionally short Si—O bonds, as highlighted in red in Tables S3 and S4, and the bond lengths in the structure model refined against the filtered structure were more chemically sensible. Regardless of the data collection sequence, energy-filtered 3D ED data consistently yielded better data-processing and structure refinement indicators.

**Figure 6:**
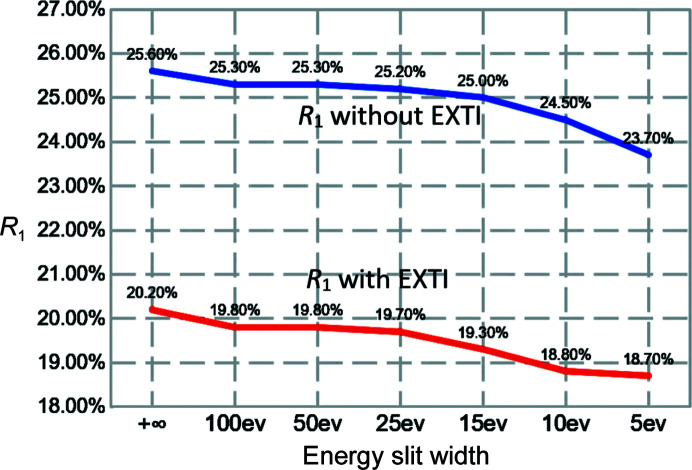
Final *R*_1_ values obtained from refinements against data sets collected on ZSM-5 (crystal 3) with various energy filter slit widths. All data sets were collected from the same crystal under identical experimental conditions. A slit width of +∞ indicates the data are unfiltered.

Furthermore, we investigated the influence of the energy slit width in energy-filtered 3D ED. We collected data sets on the same ZSM-5 crystal, denoted crystal 3, under identical experimental conditions except for the energy slit width. We first collected the unfiltered data set (slit width +∞) and then gradually decreased the width from 100 to 5 eV. Fig. 6 ▸ summarizes the final *R*_1_ values with respect to the slit width. Regardless of whether the EXTI keyword was applied, when the energy slit width is decreased, the *R*_1_ value from the structure refinement also decreased, which is consistent with the results obtained from crystals 1 and 2. In Table S5, data-processing statics such as *I*/σ and CC_1/2_ showed some minor improvements as well.

### 3.2. Comparison with other methods

There are two possible factors that contribute to the improvement of data quality. The first is the removal of inelastically scattered electrons (shown in Fig. S6), so that the data-processing software can extract the intensity of each reflection more accurately (Yonekura *et al.*, 2002 ▸, 2019 ▸). However, even without energy filtering, the low-angle reflections were not submerged in the ‘tail’ of the direct beam, unlike the case for protein crystals, in which low-angle reflections were usually overwhelmed by the central beam. Therefore, another possible reason is that inelastically scattered electrons contain some dynamically scattered electrons. When electrons interact with the crystal, some may lose energy during scattering events while others are elastically scattered. When an electron interacts with a crystal multiple times, the probability of inelastic scattering events increases. These inelastically scattered electrons produced after multiple scattering events are also removed by the energy filter, thus alleviating the influence of dynamical scattering (Yonekura *et al.*, 2015 ▸). However, we suspect that only a limited portion of the dynamically scattered electrons were removed.

To our knowledge, this is the first time that energy-filtered 3D ED data were collected using a post-column energy filter. Previously, Yonekura *et al.* (2015 ▸) and Gemmi & Oleynikov (2013 ▸) performed energy-filtered 3D ED experiments in a TEM with an in-column filter. The GIF system is much more widely available compared with the in-column filter, making the method developed in this work applicable for more TEM laboratories.

Kolb *et al*. (2019 ▸) developed automated diffraction tomography (ADT), which can also track crystals during tilting. However, their technique requires a pre-recorded STEM image tilt series from a fiducial marker or the target crystal itself. The drift of the target during tilting can then be predicted and compensated by shifting the electron beam. This technique will also require switching between a focused STEM beam and quasi-parallel STEM beam. Compared with ADT, our live STEM-HAADF tracking protocol can allow data collection over a larger tilt range. Furthermore, the method developed in this work reduces the overall electron dose and data acquisition time as no pre-recording of the TEM/STEM images was needed.

Unlike the crystal tracking method using defocused diffraction patterns (Cichocka *et al.*, 2018 ▸), the STEM-HADDF live tracking does not sacrifice any frames to form shadow images. Therefore, the completeness of the data set is higher for data collected over the same tilt range. Another advantage is that our method checks the position of the crystal almost continuously. Usually, we can adjust the scanning speed, the number of pixels and the dwelling time and hence control the acquisition time for each STEM image at 1–2 s. In contrast, the defocused diffraction pattern method will show the position of the crystal every 10 or 20 frames and the exposure time for each frame is around 0.5–2 s, as shown in Figs. S7(*a*) and S7(*b*). The operator needs to be experienced to perform crystal tracking with such a long interval since the crystal may move out of the beam before the next defocused image is displayed. This method can reliably track the crystals over large rotation intervals and will be useful for microscopes that have large *xy* movement during tilting. In addition, the crystal tracking method presented has the potential to track specific features (*e.g.* edges) for larger crystals, adding more flexibility for 3D ED data acquisition.

Currently, energy-filtered 3D ED data collection still requires an operator to re-centre the crystal according to the contrast of the HAADF image. In future work, we hope to use this method to achieve automated data collection with live crystal tracking. With increased interest in radiation-sensitive materials, a high level of automation is a way to minimize unnecessary electron dose and improve throughput of structure determination (Wang *et al.*, 2019 ▸). The crystal tracking method proposed here is generally applicable in STEM mode and can be applied to other types of camera setups.

Some challenges remain for energy-filtered 3D ED experiments. The first challenge is the distortion of the crystal lattice caused by the lenses in the energy filter. Sometimes the distortion can be so large that it is difficult to impose the correct space group during *XDS* data processing. Another challenge for energy-filtered 3D ED is finding the target crystals. As shown in Fig. S2(*b*), because of the parallel illumination, weak beam and large collection angle, when crystals are small, it is difficult to spot them in blurry HAADF images. If the camera length is too small, the collection angle will be very large (>100 mrad in our microscope), which will further decrease the contrast of the HAADF image.

### 3.3. Energy-filtered 3D ED of ZSM-5 in TEM mode

The schematic of energy-filtered 3D ED in TEM mode is shown in Fig. 1 ▸(*b*). In order to preserve the GIF alignment, we used a C3 aperture to define the illumination area on the sample instead of the selected area aperture. The size of the C3 aperture used was 30 µm, which produced a beam of 800 nm in size. One benefit of using a C3 aperture instead of an SAED aperture is to avoid unnecessary electron dose on neighbouring crystals. Since the GIF alignment can be easily affected by the settings of intermediate lenses and projection lenses, we did not re-centre the crystal during 3D ED data collection. As a result, the tilt range was limited to −40 to +40° even though the mechanical eucentric height was carefully aligned.

3D ED data were collected on the same ZSM-5 crystal over the same tilt range with and without energy filtration. The results of data processing are summarized in Table S6. The most significant improvement in data statistics is the increase of *I*/σ, from 7.74 for the unfiltered data set to 9.25 for the energy-filtered data set. In addition, CC_1/2_ slightly improved from 0.997 to 0.998.

## 4. Conclusions

In this work, we described the implementation of an energy-filtered 3D ED setup using a GIF, in both TEM mode and STEM mode. Furthermore, we proposed a live crystal tracking method using a STEM-HAADF image stream to keep the target crystal in the electron beam over a large tilt range. Using this method, it is possible to collect energy-filtered 3D ED data with a tilt range up to 150°, which is the maximum tilt range for our TEM–holder combination. In addition, we collected multiple data sets from NaCl, NH_4_H_2_PO_4_ and ZSM-5 crystals and found improvements in structure refinement using energy-filtered 3D ED data. We also found that the results of the structure refinement improve as the slit width decreases. In the case of NH_4_H_2_PO_4_, all hydrogen atoms were found and the ADPs were chemically sensible when energy-filtered data were used. The developments in this work have the potential to inspire more investigations on fine details in submicrometre-sized crystals, such as atomic motion, disorder and charge distribution.

## 5. Related literature

The following additional reference is cited in the supporting information: van Koningsveld *et al.* (1989 ▸).

## Supplementary Material

Crystal structure: contains datablock(s) NaCl_filtered_EXTI_1, NaCl_filtered_EXTI_2, NaCl_filtered_EXTI_3, NaCl_filtered_EXTI_4, NaCl_filtered_noEXTI_1, NaCl_filtered_noEXTI_2, NaCl_filtered_noEXTI_3, NaCl_filtered_noEXTI_4, NaCl_unfiltered_EXTI_5, NaCl_unfiltered_EXTI_6, NaCl_unfiltered_EXTI_7, NaCl_unfiltered_EXTI_8, NaCl_unfiltered_noEXTI_5, NaCl_unfiltered_noEXTI_6, NaCl_unfiltered_noEXTI_7, NaCl_unfiltered_noEXTI_8, NH4H2PO4_filtered_EXTI_1, NH4H2PO4_filtered_EXTI_2, NH4H2PO4_filtered_EXTI_3, NH4H2PO4_filtered_noEXTI_1, NH4H2PO4_filtered_noEXTI_2, NH4H2PO4_filtered_noEXTI_3, NH4H2PO4_unfiltered_EXTI_4, NH4H2PO4_unfiltered_EXTI_5, NH4H2PO4_unfiltered_EXTI_6, NH4H2PO4_unfiltered_noEXTI_4, NH4H2PO4_unfiltered_noEXTI_5, NH4H2PO4_unfiltered_noEXTI_6, ZSM-5_crystal3_EXTI_100ev, ZSM-5_crystal3_EXTI_10ev, ZSM-5_crystal3_EXTI_15ev, ZSM-5_crystal3_EXTI_25ev, ZSM-5_crystal3_EXTI_50ev, ZSM-5_crystal3_EXTI_5ev, ZSM-5_crystal3_EXTI_unfiltered, ZSM-5_crystal3_noEXTI_100ev, ZSM-5_crystal3_noEXTI_10ev, ZSM-5_crystal3_noEXTI_15ev, ZSM-5_crystal3_noEXTI_25ev, ZSM-5_crystal3_noEXTI_50ev, ZSM-5_crystal3_noEXTI_5ev, ZSM-5_crystal3_noEXTI_unfiltered, ZSM-5_filtered_EXTI_crystal1, ZSM-5_filtered_EXTI_crystal2, ZSM-5_filtered_noEXTI_crystal1, ZSM-5_filtered_noEXTI_crystal2, ZSM-5_unfiltered_EXTI_crystal1, ZSM-5_unfiltered_EXTI_crystal2, ZSM-5_unfiltered_noEXTI_crystal1, ZSM-5_unfiltered_noEXTI_crystal2. DOI: 10.1107/S1600576722009633/nb5321sup1.cif

Movie S1: data collection process. DOI: 10.1107/S1600576722009633/nb5321sup2.mp4

Movie S2: visual comparison between filtered/unfiltered data. DOI: 10.1107/S1600576722009633/nb5321sup3.mp4

hkl files for structure refinements. DOI: 10.1107/S1600576722009633/nb5321sup4.zip

Supporting figures and tables. DOI: 10.1107/S1600576722009633/nb5321sup5.pdf

## Figures and Tables

**Table 1:** Data-processing details using *XDS* and crystallographic details for the refinement of six filtered and unfiltered NH_4_H_2_PO_4_ data sets with and without the EXTI keyword

Data set No.	1	2	3	4	5	6
Energy filtered?	Yes	Yes	Yes	No	No	No
Rotation range (°)	144.3	137.0	140.5	130.9	140.9	143.1
Resolution (Å)	0.85	0.85	0.85	0.85	0.85	0.85
*a* (Å)	7.59	7.58	7.59	7.56	7.57	7.37
*b* (Å)	7.62	7.61	7.62	7.55	7.59	7.43
*c* (Å)	7.65	7.62	7.69	7.59	7.68	7.71
α (°)	90.44	91.14	90.72	90.69	90.82	90.68
β (°)	90.94	90.88	91.34	90.19	91.21	91.89
γ (°)	91.36	91.20	91.44	91.43	91.30	89.33
No. of reflections [*F*_o_ > 4σ(*F*_o_)]	174	166	173	171	166	155
No. of reflections (all unique)	189	183	188	179	184	183

Refinement without the EXTI keyword						
*R*_1_ [*F*_o_ > 4σ(*F*_o_)] (%)	15.7	20.4	23.5	19.7	21.9	27.2
*R*_1_ (all reflections) (%)	16.1	21.0	26.1	19.8	24.5	29.6
Goodness of fit	1.353	1.205	0.957	1.314	1.100	1.215

Refinement with the EXTI keyword						
*R*_1_ [*F*_o_ > 4σ(*F*_o_)] (%)	8.5	11.9	10.5	11.0	11.6	14.5
*R*_1_ (all reflections) (%)	8.9	12.7	12.6	11.1	14.2	18.9
Goodness of fit	1.409	1.193	1.158	1.240	1.352	1.442

**Table 2:** Data-processing details using *XDS* and crystallographic details for the refinement of eight unfiltered and filtered data sets of ZSM-5 with and without the EXTI keyword First and second in parentheses show the data collection sequence.

Crystal No.	Crystal 1		Crystal 2	
Energy filtered?	Yes (second)	No (first)	Yes (first)	No (second)
Rotation range (°)	124.2	121.0	142.8	141.9
Resolution (Å)	0.90	0.90	0.90	0.90
Completeness (%)	84.5	82.4	86.3	85.8
*I*/σ	4.34	3.53	4.16	4.03
CC_1/2_ (%)	99.6	98.8	99.0	98.8
Observed reflections	12546	12439	17038	16984
*R*_meas_ (%)	15.9	22.5	13.5	13.7
Reflections [*F*_o_ > 4σ(*F*_o_)]	1775	1702	2397	2327
No. of reflections (all unique)	3354	3279	3438	3429

Refinement without EXTI keyword				
*R*_1_ [*F*_o_ > 4σ(*F*_o_)] (%)	27.5	30.0	25.1	26.6
*R*_1_ (all reflections) (%)	31.7	34.4	27.5	29.3
Goodness of fit	1.074	1.093	2.132	1.950
No. of NADP/splitting atoms	3	32	0	1

Refinement with EXTI keyword				
*R*_1_ [*F*_o_ > 4σ(*F*_o_)] (%)	24.3	26.4	19.7	23.3
*R*_1_ (all reflections) (%)	28.7	30.6	22.1	24.4
Goodness of fit	1.740	1.619	1.001	1.554
No. of NADP/splitting atoms	0	2	0	0
