# Supplementary material for: Improving data quality for three-dimensional electron diffraction by a post-column energy filter and a new crystal tracking method
Source: J Appl Crystallogr. 2022 Nov 29;55(Pt 6):1583–91. doi: 10.1107/S1600576722009633 (PMC9721325; doi:10.1107/S1600576722009633)
Supplement: Supplementary file 5 [file j-55-01583-sup5.pdf]

JOURNAL OF APPLIED CRYSTALLOGRAPHY

Volume 55 (2022)

Supporting information for article:

Improving data quality for three-dimensional electron diffraction by a post-column energy filter and a new crystal tracking method

Taimin Yang, Hongyi Xu and Xiaodong Zou

Improving data quality for three-dimensional electron diffraction by a post-column energy filter and a new crystal tracking method

Taimin Yang[a*], Hongyi Xu[a*], Xiaodong Zou[a]

a Department of Materials and Environmental Chemistry (MMK), Stockholm University, Svante Arrhenius väg 16C, Stockholm, SE-10691, Sweden

* Corresponding email: taimin.yang@mmk.su.se hongyi.xu@mmk.su.se

Table of Contents **Page**

Supporting Figures…………………………………………………………………… S2

Supporting Tables..…………………………………………………………………... S9

Supplementary Movies………………………………………………………………. S17

References…………………………………………………………………………..... S18

List of Tables and Figures

Figure S1: Schematics of the crystal tracking using STEM-HAADF images.

Figure S2: Images taken from the same area under (a) TEM mode (b) STEM mode.

Figure S3: Typical HAADF images at high magnification during 3DED data collection.

Figure S4: Typical structure of NaCl refined (a) with EXTI keyword (b) without EXTI keyword.

Figure S5: Normalized scaling factors from tilt series collected for three $NH_4H_2PO_4$ crystals.

Figure S6: Comparison between energy filtered and unfiltered ED patterns.

Figure S7: Comparison between different crystal tracking method.

Table S1: Data processing details using XDS and crystallographic details for the refinement for eight datasets of NaCl collected in STEM mode with and without EXTI keyword.

Table S2: Deviations of atomic positions between the reference ZSM-5 structure (van Koningsveld *et al.*, 1987) and those determined from filtered and unfiltered 3DED data collected from crystal 1.

Table S3: Refined Si-O bond distances for crystal 1 of ZSM-5 structure from filtered and unfiltered and the reference structure.

Table S4: Refined Si-O bond distances for crystal 2 of ZSM-5 structure from filtered and unfiltered and the reference structure.

Table S5: Data processing details using XDS and crystallographic details for the refinement for datasets collected from ZSM-5 crystal 3

Table S6: Data processing details using XDS for two datasets of ZSM-5 collected in TEM mode with and without energy filtration

Supplementary Figures

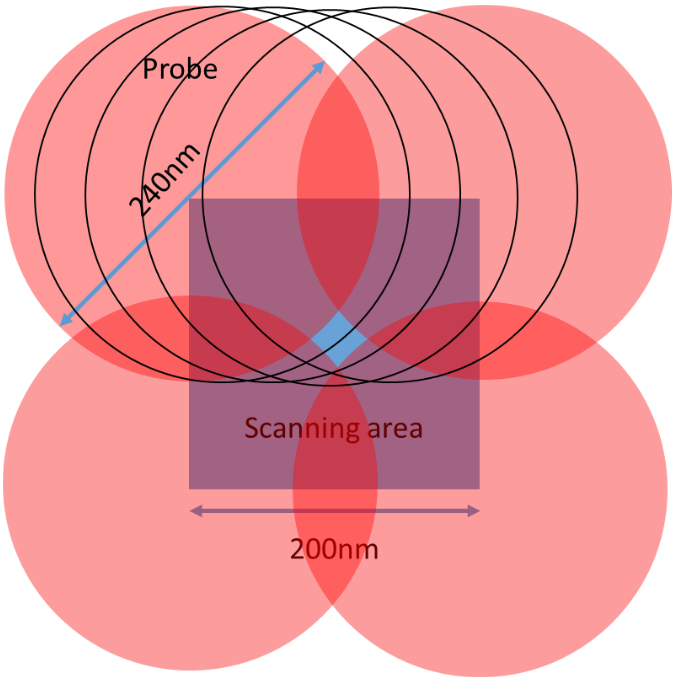

Figure S1. Schematics of the beam setup used for continues rotation 3DED data collection and crystal tracking by STEM-HAADF. The scanning of the electron beam allows the formation of the low resolution HAADF image, while 3DED data is collected over the entire scanned area.

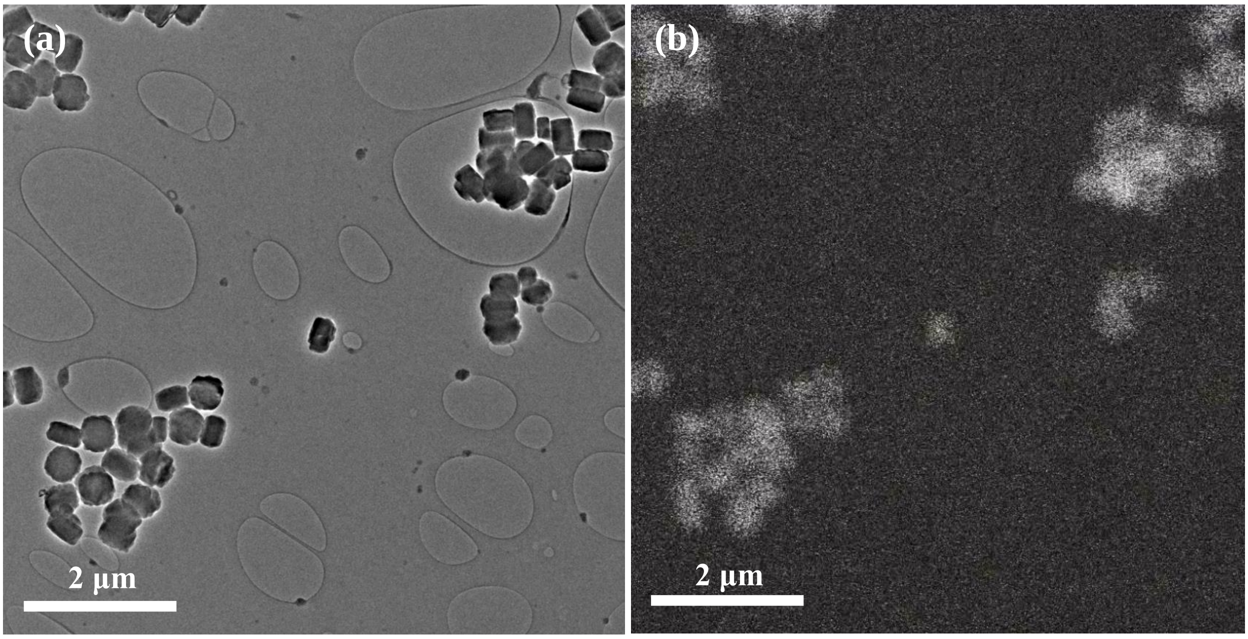

Figure S2. Images taken from the same area in (a) TEM mode (b) STEM mode with a parallel beam. In the HAADF-STEM image, the bright blobs are target crystals.

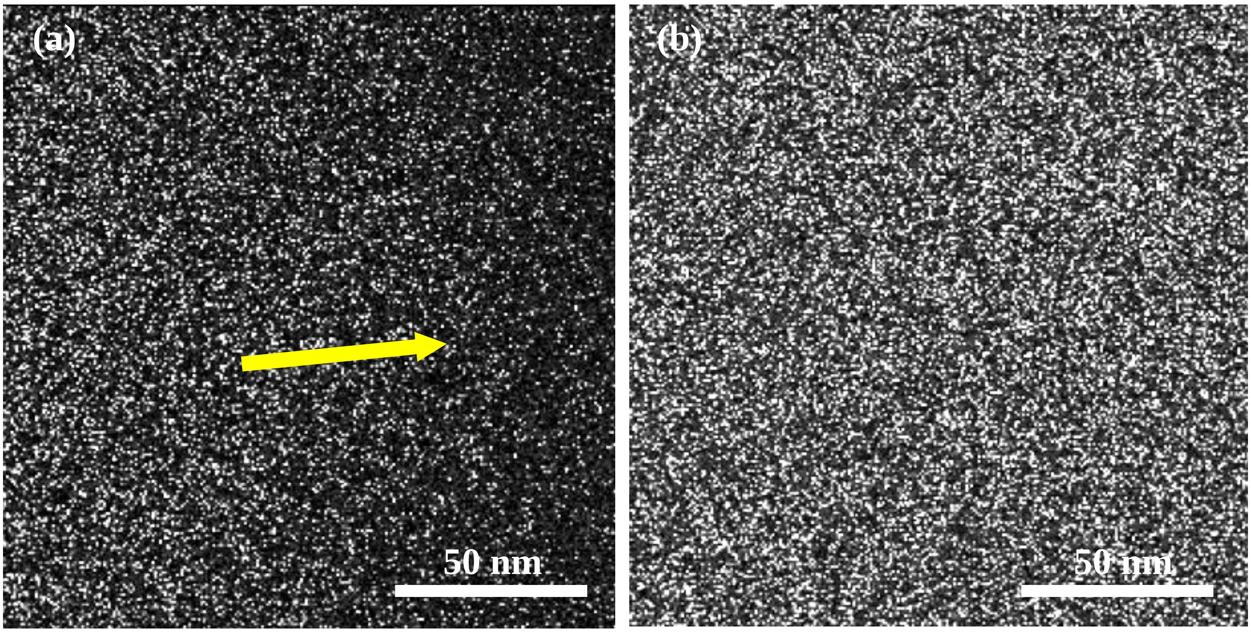

Figure S3. Typical HAADF images of ZSM-5 sample at high magnification during 3DED data collection. (a) An example when scanning area was located at the edge of the target crystal. The position of the darker area in the image indicates the stage adjustment direction, as shown by the blue arrow. (b) After adjusting the stage position, the scanning area moved back to the center part of the crystal.

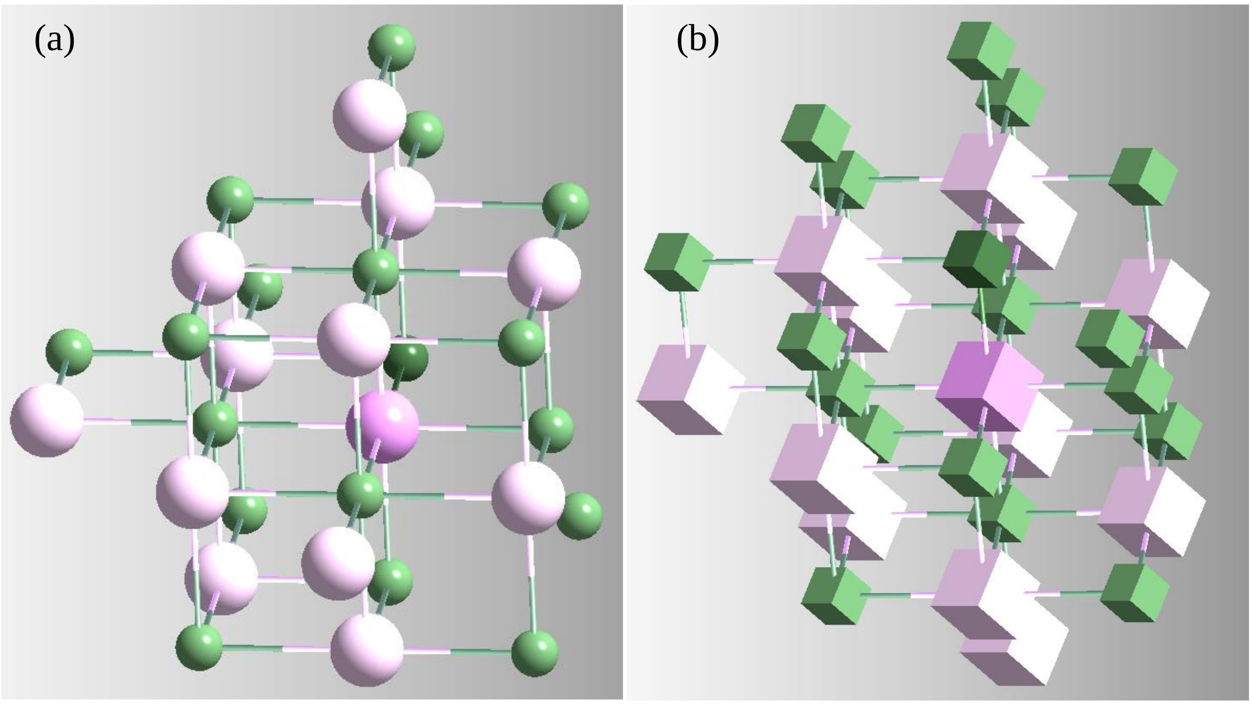

Figure S4. Typical structure of NaCl refined (a) with EXTI keyword (b) without EXTI keyword

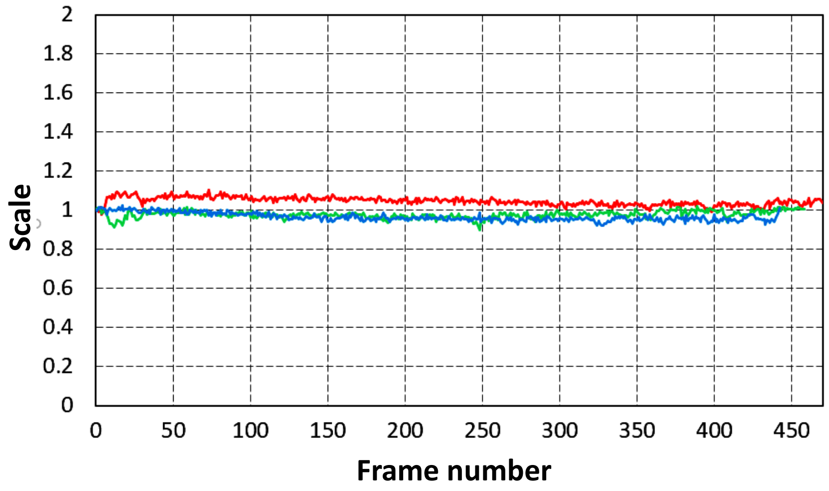

Figure S5. Normalized scaling factors from diffraction patterns collected for three $NH_4H_2PO_4$ crystals as calculated by XDS (SCALE in file INIT.Lp) can be used to judge the effectiveness of the crystal tracking method. If the crystal moves (partially) out of the electron beam or selected area aperture, the image scale will be affected and deviate from 1(Cichocka *et al.*, 2018).

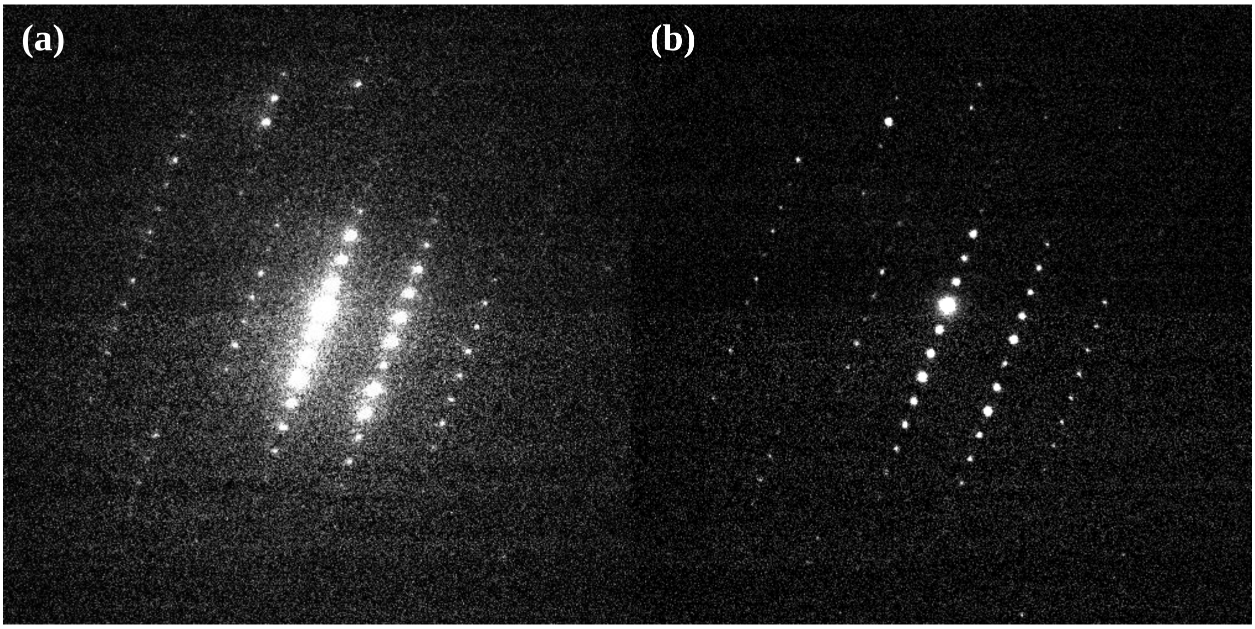

Figure S6. Typical electron diffraction patterns of ZSM-5 crystal (a) without energy filtration and (b) with energy filtration. The profiles of the reflections in the filtered electron diffraction pattern were much sharper.

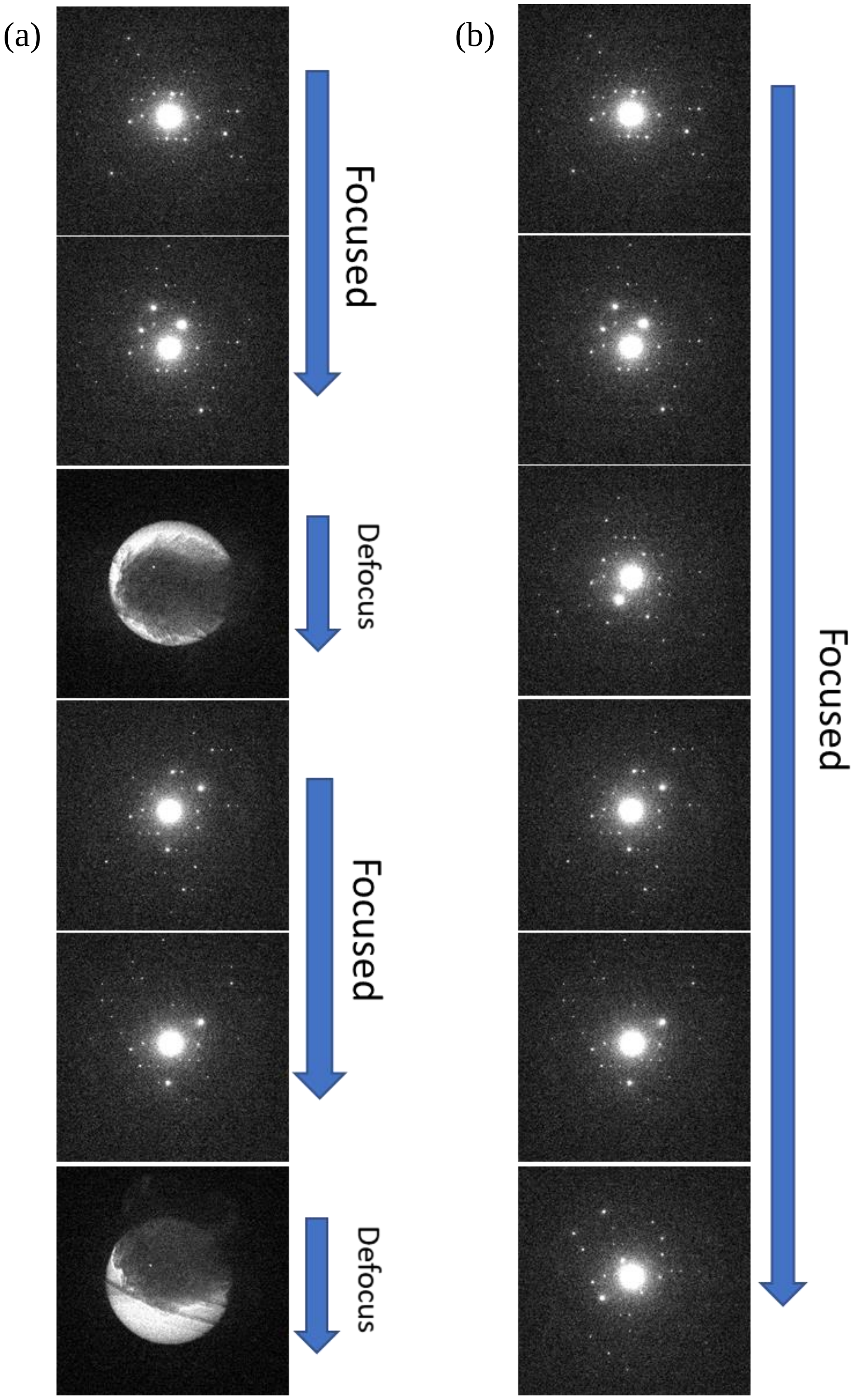

Figure S7. Comparison between different crystal tracking methods (a) defocus diffraction pattern tracking (b) live STEM-HAADF imaging tracking

Supplementary Tables

Table S1. Data processing details using XDS and crystallographic details for the refinement for eight datasets of NaCl collected in STEM mode with and without EXTI keyword.

**Dataset no.**	**1**	**2**	**3**	**4**	**5**	**6**	**7**	**8**
Energy-filtered	Yes	Yes	Yes	Yes	No	No	No	No
Rotation Range (°)	137.2	150.4	150.6	133.4	136.1	144.3	135.2	137.9
Resolution (Å)	0.8	0.8	0.8	0.8	0.8	0.8	0.8	0.8
No. refl (F_o>4sig(F_o))	21	19	21	21	21	18	19	16
No. all unique refl	21	20	21	21	21	19	20	18
**Refinement without EXTI keyword**								
R_1 (F_o > 4sig(F_o))	32.2%	21.0%	23.6%	20.8%	33.5%	19.8%	23.5%	32.6%
R_1 (all reflections)	32.2%	21.2%	23.6%	20.8%	33.5%	19.8%	24.2%	30.8%
Goof	1.026	1.432	1.276	1.242	1.076	1.323	1.322	2.151
**Refinement with EXTI keyword**								
R_1 (F_o > 4sig(F_o))	7.9%	7.5%	10.1%	8.3%	11.7%	13.8%	15.6%	14.7%
R_1 (all reflections)	7.9%	7.9%	10.1%	8.3%	11.7%	15.2%	15.7%	15.9%
Goof	1.34	1.461	1.464	1.217	1.34	1.383	1.481	1.038

Table S2. Deviations of atomic positions between the reference ZSM-5 structure (van Koningsveld *et al.*, 1987) and those determined from filtered and unfiltered 3DED data from crystal 1. Fractional atomic coordinates for the reference ZSM-5 structure determined by SCXRD (as-made ZSM-5, space group *Pnma*, a = 20.022(4) Å, b = 19.899(4) Å, c = 13.383(3) Å, see the International Zeolite Association (IZA) Database).

Atom	Atom position deviation, unfiltered, Å	Atom position deviation, filtered, Å
Si1	0.025	0.028
Si2	0.036	0.049
Si3	0.061	0.033
Si4	0.055	0.019
Si5	0.051	0.040
Si6	0.048	0.018
Si7	0.057	0.066
Si8	0.044	0.023
Si9	0.043	0.025
Si10	0.061	0.058
Si11	0.025	0.046
Si12	0.066	0.023
O1	0.088	0.092
O2	0.070	0.031
O3	0.050	0.070
O4	0.119	0.104
O5	0.030	0.068
O6	0.128	0.088
O7	0.102	0.045
O8	0.127	0.146
O9	0.053	0.031
O10	0.135	0.124
O11	0.051	0.115
O12	0.140	0.147
O13	0.093	0.045
O14	0.040	0.069
O15	0.116	0.103
O16	0.079	0.110
O17	0.086	0.055
O18	0.102	0.046
O19	0.031	0.073
O20	0.115	0.022

O21	0.087	0.076
O22	0.089	0.152
O23	0.092	0.064
O24	0.088	0.040
O25	0.028	0.046
O26	0.058	0.065
**<Si> average**	0.048(13)	0.035(15)
**<O> average**	0.085(33)	0.078(37)

Table S3. Refined Si-O bond distances for crystal 1 of ZSM-5 structure from filtered and unfiltered and the reference structure(van Koningsveld *et al.*, 1987). The number marked with red color means severe deviation from reference bond length.

Atomic 1	Atomic 2	Bond length unfiltered, Å	Bond length filtered, Å	Bond length ref, Å
SI1	O1	1.6091	1.5863	1.5830
SI1	O15	**1.4953**	1.6079	1.5914
SI1	O16	1.5976	1.5778	1.5800
SI1	O21	1.6438	1.6188	1.5977
SI2	O1	1.6031	1.5701	1.5867
SI2	O2	1.6261	1.5654	1.6011
SI2	O6	1.5593	1.6620	1.5816
SI2	O13	**1.4943**	1.6422	1.5676
SI3	O2	1.6064	1.6198	1.5867
SI3	O3	1.6068	1.5706	1.5708
SI3	O19	1.5732	1.5021	1.5711
SI3	O20	1.5144	1.6056	1.5914
SI4	O3	1.5355	1.5813	1.5748
SI4	O4	1.6345	1.6661	1.5861
SI4	O16	1.5596	1.6203	1.5825
SI4	O17	1.5809	1.6421	1.5889
SI5	O4	1.5530	1.5016	1.5829
SI5	O5	1.5774	1.5436	1.5891
SI5	O14	1.5709	1.6330	1.5825
SI5	O21	1.5547	1.5113	1.5980
SI6	O5	1.6274	1.6686	1.5942
SI6	O6	1.6007	1.5377	1.5879
SI6	O18	1.5713	1.6239	1.5935
SI6	O19	1.5528	1.6236	1.5855
SI7	O7	1.5626	1.5221	1.5804
SI7	O17	1.5816	1.5108	1.5860
SI7	O23	1.5947	1.6347	1.5849
SI7	O22	1.5683	1.5132	1.5905
SI8	O7	1.5931	1.5921	1.5856
SI8	O8	1.5791	1.5685	1.5882
SI8	O12	**1.6819**	1.5776	1.5827
SI8	O13	1.6344	1.5172	1.5765
SI9	O8	1.6201	1.6187	1.5783
SI9	O9	1.5844	1.6015	1.5909
SI9	O25	1.5591	1.5917	1.5983
SI9	O18	1.6628	1.5921	1.5971
SI10	O9	1.6285	1.6096	1.5897
SI10	O10	1.6081	1.5967	1.5730

SI10	O26	1.6259	1.6017	1.6049
SI10	O15	1.6313	1.5471	1.5884
SI11	O10	1.5329	1.5724	1.5809
SI11	O11	1.5983	1.6361	1.5910
SI11	O14	1.5788	1.5588	1.5681
SI11	O22	1.5560	1.6798	1.5937
SI12	O11	1.6006	1.5055	1.5857
SI12	O12	**1.4469**	1.5580	1.5742
SI12	O20	1.6148	1.6054	1.6055
SI12	O24	1.6086	1.6240	1.5952
**Average Length**		**1.5863(476)**	**1.5868(442)**	**1.5866(88)**

Table S4. Refined Si-O bond distances for crystal 2 of ZSM-5 structure from filtered and unfiltered and the reference structure(van Koningsveld *et al.*, 1987). The number marked with red color means severe deviation from reference bond length.

Atomic 1	Atomic 2	Bond length unfiltered, Å	Bond length filtered, Å	Bond length ref, Å
SI1	O1	1.609	1.592	1.5830
SI1	O15	1.587	1.611	1.5914
SI1	O16	1.581	1.571	1.5800
SI1	O21	1.598	1.561	1.5977
SI2	O1	1.546	1.562	1.5867
SI2	O2	1.568	1.552	1.6011
SI2	O6	1.582	1.582	1.5816
SI2	O13	1.558	1.592	1.5676
SI3	O2	1.610	1.611	1.5867
SI3	O3	1.599	1.601	1.5708
SI3	O19	1.625	1.601	1.5711
SI3	O20	1.597	1.612	1.5914
SI4	O3	1.557	1.551	1.5748
SI4	O4	1.573	1.571	1.5861
SI4	O16	1.586	1.571	1.5825
SI4	O17	1.570	1.581	1.5889
SI5	O4	1.588	1.581	1.5829
SI5	O5	1.582	1.591	1.5891
SI5	O14	1.569	1.631	1.5825
SI5	O21	1.574	1.601	1.5980
SI6	O5	1.631	1.632	1.5942
SI6	O6	1.581	1.602	1.5879
SI6	O18	1.656	1.621	1.5935
SI6	O19	1.555	1.581	1.5855
SI7	O7	1.615	1.592	1.5804
SI7	O17	1.601	1.581	1.5860
SI7	O23	1.593	1.595	1.5849
SI7	O22	1.600	1.581	1.5905
SI8	O7	1.519	1.561	1.5856
SI8	O8	1.591	1.601	1.5882
SI8	O12	1.621	1.541	1.5827
SI8	O13	1.595	1.582	1.5765
SI9	O8	1.571	1.571	1.5783
SI9	O9	1.603	1.591	1.5909
SI9	O25	1.621	1.607	1.5983
SI9	O18	1.513	1.551	1.5971
SI10	O9	1.600	1.601	1.5897
SI10	O10	1.572	1.521	1.5730

SI10	O26	1.610	1.592	1.6049
SI10	O15	1.582	1.581	1.5884
SI11	O10	1.557	1.621	1.5809
SI11	O11	1.581	1.572	1.5910
SI11	O14	1.583	1.541	1.5681
SI11	O22	1.542	1.591	1.5937
SI12	O11	1.580	1.592	1.5857
SI12	O12	1.603	1.601	1.5742
SI12	O20	1.508	1.552	1.6055
SI12	O24	1.600	1.598	1.5952
**Average Length**		**1.5842(289)**	**1.5850(238)**	**1.5866(88)**

Table S5. Data processing details using XDS and crystallographic details for the refinement for datasets collected from ZSM-5 crystal 3 with and without EXTI keyword. The rotation range, resolution and completeness for each dataset was kept at 130°, 0.9Å and 69.6%, respectively. Slit width of $+\infty$ means the data is unfiltered.

**Slit width**	$+\infty$	**100ev**	**50ev**	**25ev**	**15ev**	**10ev**	**5ev**
I/SIGMA	3.86	4.10	4.12	4.31	4.25	4.21	4.42
CC(1/2) (%)	98.8	98.8	98.9	99.4	98.9	99.2	99.3
Observed Reflections	14472	14453	14515	14639	14639	14774	14670
R-meas (%)	16.7	15.8	15.2	15.2	15.4	15.7	15.0
No. of reflections ($F_o > 4sig(F_o)$)	1782	1925	1834	1886	1860	1869	1875
No. of reflections (all unique)	2797	2787	2801	2796	2782	2836	2794
**Refinement without EXTI keyword**							
R_1 (> 4sig(F_o))	25.6%	25.3%	25.3%	25.2%	25.0%	24.5%	23.7%
R_1 (all)	29.1%	28.0%	28.2%	27.8%	27.7%	27.4%	26.4%
**Refinement with EXTI keyword**							
R_1 (> 4sig(F_o))	20.2%	19.8%	19.8%	19.7%	19.3%	18.8%	18.7%
R_1 (all)	23.7%	22.4%	22.5%	22.0%	21.7%	21.4%	21.2%

Table S6. Data processing details using XDS for two datasets of ZSM-5 collected in TEM mode with and without energy filtration

	Unfiltered	Energy-filtered
Observed Reflections	9199	9920
I/SIGMA	7.74	9.25
CC(1/2)	99.7	99.8
Rotation Range (°)	64.6	69.8
Resolution (Å)	0.9	0.9
a (Å)	19.76	19.75
b (Å)	19.73	19.67
c (Å)	13.66	13.67
α (°)	91.57	91.00
β (°)	90.85	90.23
γ (°)	90.32	91.68

Supplementary Movies

Movie S1. Demonstration of the HAADF image stream crystal tracking method.

Movie S2. Comparison between 3DED datasets with energy filtering and without filtering for the same ZSM-5 crystal.

References

Cichocka, M. O., Ångström, J., Wang, B., Zou, X. & Smeets, S. (2018). *J Appl Cryst*. **51**, 1652–1661.

van Koningsveld, H., van Bekkum, H. & Jansen, J. C. (1987). *Acta Cryst B*. **43**, 127–132.
